# Low-dose aspirin protective effects are correlated with deregulation of HNF factor expression in the preeclamptic placentas from mice and humans

**DOI:** 10.1038/s41420-019-0170-x

**Published:** 2019-05-10

**Authors:** Aurélien Ducat, Alexandra Vargas, Ludivine Doridot, Alessia Bagattin, Jonathan Lerner, Jean-Luc Vilotte, Christophe Buffat, Marco Pontoglio, Francisco Miralles, Daniel Vaiman

**Affiliations:** 1 Institut Cochin, INSERM U1016, UMR 8104 CNRS, Faculté René Descartes, 24 rue du Faubourg St Jacques, 75014 Paris, France; 2 Epigenetics and Cell Signaling, Institute for Advanced Biosciences, Inserm U1209, CNRS UMR 5309, Université Grenoble Alpes, 38000 Grenoble, France; 3 GABI, INRA, AgroParisTech, Université Paris-Saclay, 78352 Jouy-en-Josas, France; 4 Department of Neonatology, Hôpital La Conception, 147 Boulevard Baille, 13005 Marseille, France

**Keywords:** Cardiovascular biology, Transcriptomics

## Abstract

Aspirin (acetyl-salicylic acid) is one of the most ancient drugs of the human pharmacopeia. Nonetheless, its action at low doses is not well understood at the molecular level. One of the applications of low-dose aspirin treatment is the prevention of preeclampsia (PE) in patients at risk. Foeto-placental overexpression of the STOX1A transcription factor in mice triggers PE symptoms. Transcriptomic analysis of the placentas, showed that aspirin massively down-regulates genes of the coagulation and complement cascade, as well as genes involved in lipid transport. The genes modified by aspirin treatment are not the ones that are modified by STOX1 overexpression, suggesting that aspirin could act downstream, symptomatically on the preeclamptic disease. Bioinformatics analysis of the promoters of the deregulated genes showed that they are strongly enriched in HNF transcription factors-binding sites, in accordance with existing literature showing their roles as regulators of coagulation. Two of these transcription factors, *Hnf1β* and *Hnf4α* are found down-regulated by aspirin treatment. In parallel, we show that in human patient placentas, aspirin-induced deregulations of genes of the coagulation cascade are also observed. Finally, the expression of Hnf1β target sequences (*Kif12*, *F2*, *Hnf4α* promoters and a synthetic concatemer of the Hnf1β-binding site) were investigated by transfection in trophoblast cell models, with or without aspirin treatment and with or without STOX1A overexpression. In this model we observed that STOX1A and aspirin tended to synergize in the down-regulation of Hnf1β target genes in trophoblasts.

## Introduction

Preeclampsia (PE) is a major disease of pregnancy characterized by the occurrence of de novo hypertension and proteinuria in pregnant women from the 20th week of gestation^1^. Once diagnosed, it can only progress towards a worsening of the symptoms, leading sometimes to the extraction of the fetus to preserve the mother health. Thus, PE is a major contributor of prematurity^2^, which in turn, is an important risk factor for programming diseases in adult life^3^. Treatments against PE are limited. Meta-analyses of randomized control trials show that low-dose aspirin (75–150 mg/day) reduces the incidence of PE by ~10–30%^4,5^. This effect is much higher when aspirin is given before 16 weeks of pregnancy^6–9^. The molecular effects of aspirin at low-doses are not well understood. Aspirin has anti-inflammatory, anti-thrombotic, and anti-oxidant pharmacological properties, and as such, could act by preventing the systemic endothelial dysfunction characteristic of PE. Besides, aspirin could have direct effects upon the establishment of early placental circulation consistently with its protective action against PE being essentially visible at early terms.

The understanding of the prophylactic action of aspirin, can be approached using animal models of PE. Recently, we developed a model of severe PE in mice induced by the foeto-placental overexpression of the human transcription factor STOX1A^10^. In this model, we observed that the complete set of symptoms can be corrected by the administration of aspirin in the drinking water at a dose corresponding to the equivalent of 150–200 mg/day in humans. Here, we compared placental gene expression with or without aspirin treatment in control and PE mice. Bioinformatics analysis revealed that, aspirin specifically and dramatically down-regulates genes of the coagulation cascade as well as genes involved in lipid transport. However, aspirin did not target genes that are modified in the placenta by STOX1A overexpression. The analysis of the promoters of the aspirin-deregulated genes showed that they are strongly enriched in HNF transcription factors-binding sites. In the mouse placentas, we show that STOX1A overexpression decreases the protein level of Hnf1β. In human placentas, the transcripts levels of HNF1β targets were also decreased. We also studied in vitro (in a JEG-3 cells) the effects of STOX1A and aspirin on the promoters of HNF1β targets, indicating that in this model at least, STOX1A induces rather a down-regulation. This could imply that STOX1A does not play a noxious role by increasing coagulation gene expression. Overall, our results suggest that the prophylactic effects of aspirin in the placenta result in a decrease of the expression of coagulation genes.

## Results

### Low-dose aspirin treatment down-regulates coagulation genes in STOX1A-overexpressing placentas

**Fig. 1:**
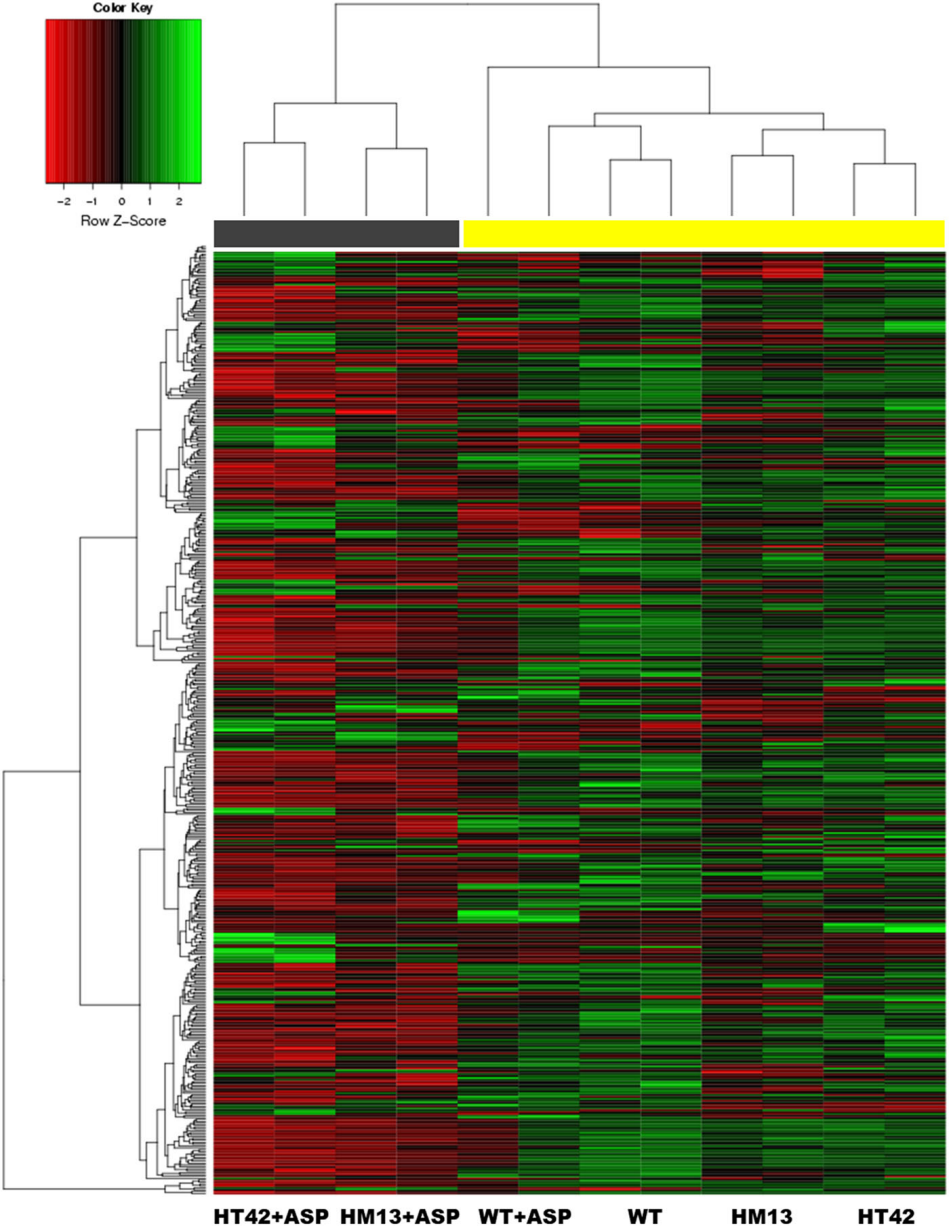
Semi-supervised clustering presented as a heatmap representation of the 500 most significant genes selected through their increasing *p-*value by Arraymining (see the section “Material and methods”). Each lane is the result of the hybridization of a pool of three placentas. The induction ratios were analyzed by ANOVA. HM13 and HT42 refer to two strains of transgenic mice overexpressing STOX1, a model for preeclampsia^10^. Transgenic placentas from aspirin-treated mice (black bar) are separated from the others (yellow bar)

RNA from placental samples was prepared and hybridized to Nimblegen mouse expression arrays. These samples corresponded to placentas, obtained by crossing WT ♀ mice (receiving or not aspirin treatment), either with WT or transgenic STOX1 ♂ mice^10,11^. The differentially expressed genes (DEGs) between groups were sorted using Arraymining (http://www.arraymining.net/R-php-1/ASAP/microarrayinfobiotic.php) by increasing ANOVA *p*-values. Figure 1 shows the 500 genes with the lowest *p*-values, which allowed a semi-supervised clustering of transcripts and samples. This divided the samples into two groups: a group including exclusively the transgenic placentas of aspirin-treated mice (marked with a gray box). The other group corresponded to the other placentas, including the controls treated or not with aspirin. Thus, the variation induced by aspirin treatment alone or by STOX1 overexpression alone is small compared to the one induced by the combination Aspirin+STOX1 overexpression. Overall, transgenic placenta from aspirin-treated mice were markedly enriched in down-regulated genes (all the genes in red in the four left columns of Fig. 1). In sum, the genes presented in the heatmap in the context “PE+Aspirin” (i.e. HT42+ASP and HM13+ASP) drive the largest part of the variance, suggesting a strong effect of aspirin treatment, but only when the placentas over-express STOX1A. This was further analyzed by a WebGestalt analysis of the gene clusters modified by Aspirin in terms of gene ontology (Table 1). Comparing transgenic placentas from aspirin-treated versus transgenic placentas from non-aspirin-treated mice, the main enriched pathway (using KEGG or Reactome databases) was by far “Complement and Coagulation Cascades” (KEGG: FDR ≤ 10^−20^; Reactome: FDR ≤ 4.8 × 10^−7^). In the WT placentas, the aspirin treatment enriched primarily the same gene cascade but with a much lower statistical significance (FDR ≤ 1.52 × 10^−2^, Table 1). In the PE aspirin-treated placentas, other pathways associated with lipid metabolism and liposoluble vitamins, were found (such as “PPAR signaling”, “fat digestion and absorption”, “metabolism of fat soluble vitamins”, “vitamin digestion and absorption”). These differential enrichments clearly revealed that the aspirin effects depend upon the genetic context.

**Table 1:** KEGG and Reactome pathways of down-regulated genes under aspirin treatment (<two fold)

Comparison	Pathway (KEGG & Reactome)	Genes	FDR
STOX+ASP vs. STOX	Complement and coagulation cascades	22	<10−15
	Drug metabolism—cytochrome P450	9	1.64 × 10^−3^
	Vitamin digestion and absorption	5	8.50 × 10^−3^
	PPAR siganling pathway	8	8.50 × 10^−3^
	Fat digestion and absorption	6	1.08 × 10^−2^
	Metabolism of fat-soluble vitamins	14	1.66 × 10^−9^
	Common pathways of fibrin clot formation	9	1.44 × 10^−7^
	Retinoid metabolism and transport	11	2.94 × 10^−7^
	Complement cascade	11	4.84 × 10^−7^
	Lipoprotein metabolism	11	4.59 × 10^−5^
WT+ASP vs. WT	Complement and coagulation cascades	7	1.52 × 10^−2^
	ECM–receptor interaction	6	7.66 × 10^−2^
	Mineralocorticoid biosynthesis	3	5.86 × 10^−2^

### Genes modified by aspirin are not superposed to genes modified by STOX1A overexpression in the transgenic placentas

**Fig. 2:**
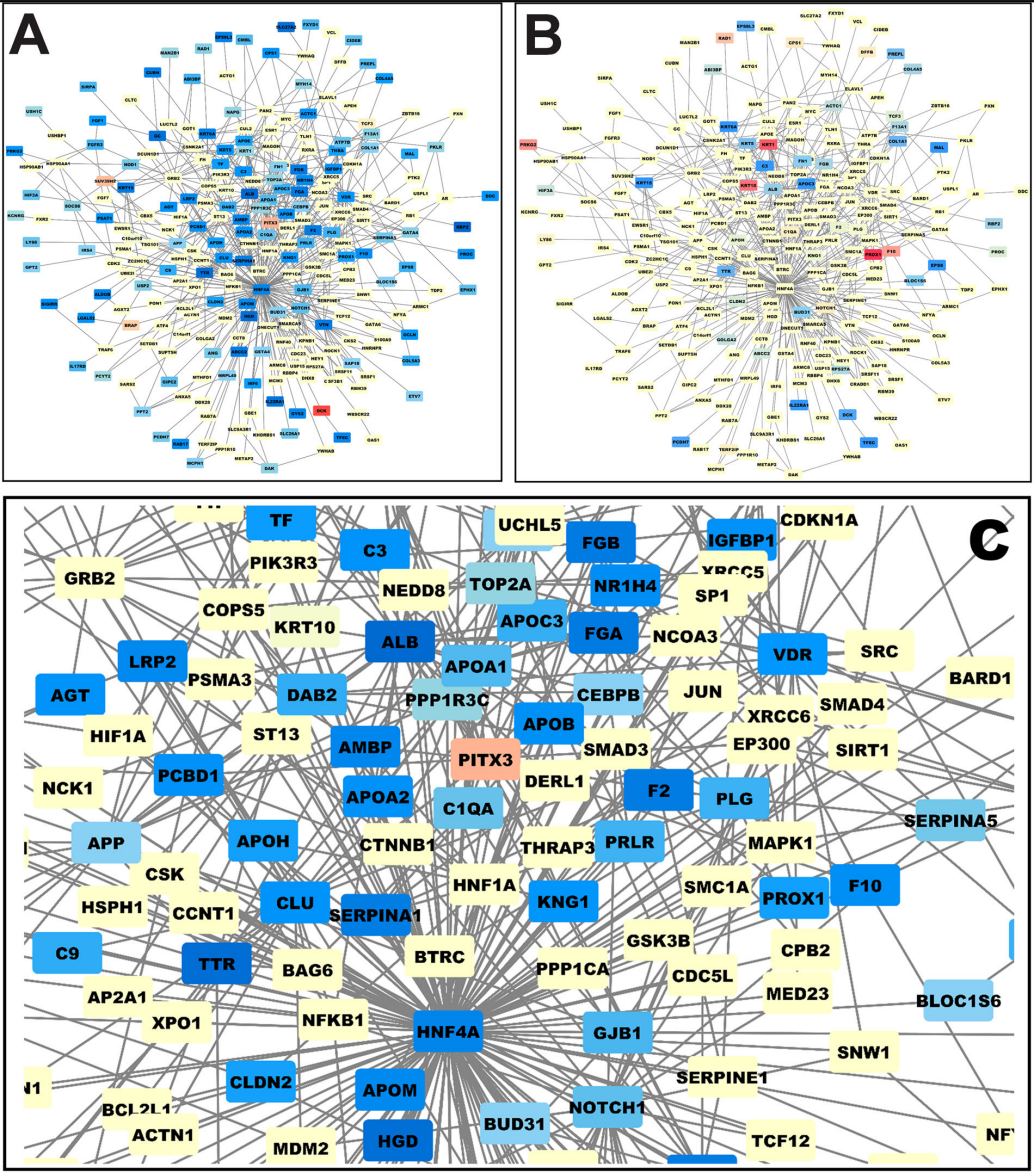
The genes that are deregulated in the placenta are connected in a functional network of genes modified in the placenta. The colors mark genes that are deregulated (red for up-regulated and blue for down-regulated in this graph). **a** A dense sub-network of down-regulated genes appears. The same network of genes is presented in **b**, but this time, the colors correspond to the genes that are deregulated by STOX1 overexpression in the placenta. **c** A zooming from (**a**) with the genes labeled in blue in the network, enabling to read their names (note in particular the down-regulation of the thrombin gene, the F2 coagulation factor)

**Fig. 3:**
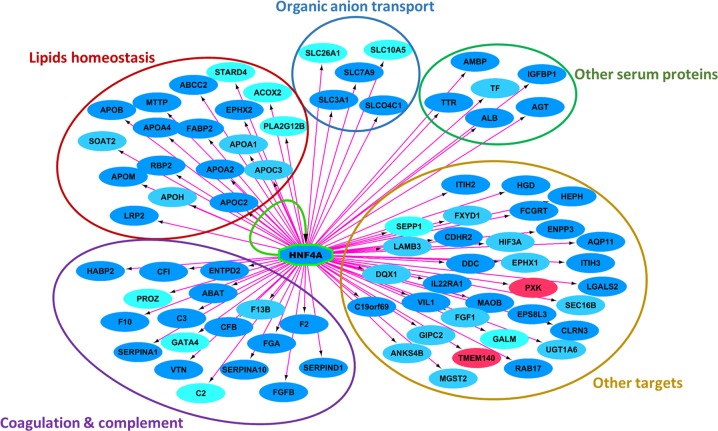
We used the cytoscape add-in iRegulon to analyze systematically the composition of the gene promoters in transcription factor-binding sites. The genes in blue are down-regulated by aspirin treatment, while the genes in red are up-regulated. The pink arrows indicate that at least one HNF4α-binding site is present in the promoter of the genes. Genes are grouped according to biological functions, showing that HNF4α transcription factor-binding sites are over-represented in the promoter of many of the genes that are down-regulated in the placenta following aspirin treatment. HNF4α itself is strongly down-regulated in the placenta by aspirin treatment, and the yellow arrow indicate a feedback loop of regulation

We performed a network analysis of the DEGs using the String and Cytoscape softwares (https://string-db.org/ and https://cytoscape.org/. Figure 2a presents the network of genes deregulated by aspirin treatment (down-regulated genes in blue and up-regulated genes in red). A majority of genes are down-regulated by aspirin treatment. Figure 2b shows the same network but labeling in red and blue the genes deregulated by STOX1A in the absence of aspirin. Clearly, the colors cannot be superposed meaning that STOX1A does not alter the expression of the same genes than aspirin treatment. Thus, STOX1A deregulates genes in the placenta triggering a preeclamptic phenotype, while aspirin deregulates a completely different set of genes. Further, we analyzed the specific group of genes down-regulated by aspirin. Zooming on this cluster of genes (Fig. 2c) revealed that most of them are involved in regulating coagulation and complement as shown in the KEGG pathway (Supplementary Fig. 1). The network analysis using Cytoscape positions HNF4α in the center of the modified genes. The level of deregulation and statistics for these genes is presented as Supplemental Table 2. Using the iRegulon add-in of Cytoscape we analyzed the promoters of these down-regulated genes and detected that they are significantly enriched (normalized enrichment score = 6.69; *p* < 0.0001) in binding sites for transcription factors of the HNF family (HNF1α, HNF1β, HNF4α, and HNF4γ). Functional ontology of these genes showed that they were involved in coagulation and complement, lipid homeostasis, organic ion transport, plasmatic/serum proteins, and other miscellaneous targets (Fig. 3).

### HNF1β expression is reduced in transgenic placentas treated by Aspirin

We used our microarray analysis to examine the relative expression of HNF genes in the placentas (Table 2). The expression was low for *Hnf1α*, taken as a reference (arbitrarily fixed to 1), very low for *Hnf4γ* (0.31), medium for *Hnf1β* (3.9), and very high for *Hnf4α* (14.2). At the mRNA level, *Hnf4α* mRNA abundance was decreased by 3.15 fold in the transgenic placentas of aspirin-treated mice (*Hnf1α* was reduced by only 13%, and *Hnf1β* was decreased by 27%).

**Table 2:** Aspirin effect on HNF genes in transgenic and non-transgenic placentas

	Expression level	Aspirin effect in WT placentas	Aspirin effect in STOX1 transgenic placentas	STOX1 effects	Overall aspirin effect	Interaction effect
*Hnf1α*	Low (1)	1.444	0.870	0.997	ns	ns
*Hnf1β*	Medium (3.9)	**0.705**	**0.728**	0.757	*p* = 0.007	ns
*Hnf4α*	High (14.2)	**0.614**	**0.317**	**0.488 (*p*** **=** **0.013)**	*p* = 0.0002	*p* = 0.0088
*Hnf4γ*	Low (0.31)	1.444	0.977	1.025	ns	ns

Bold values stand for cases where significance was observed in at least one comparison (*P* < 0.05)

**Fig. 4:**
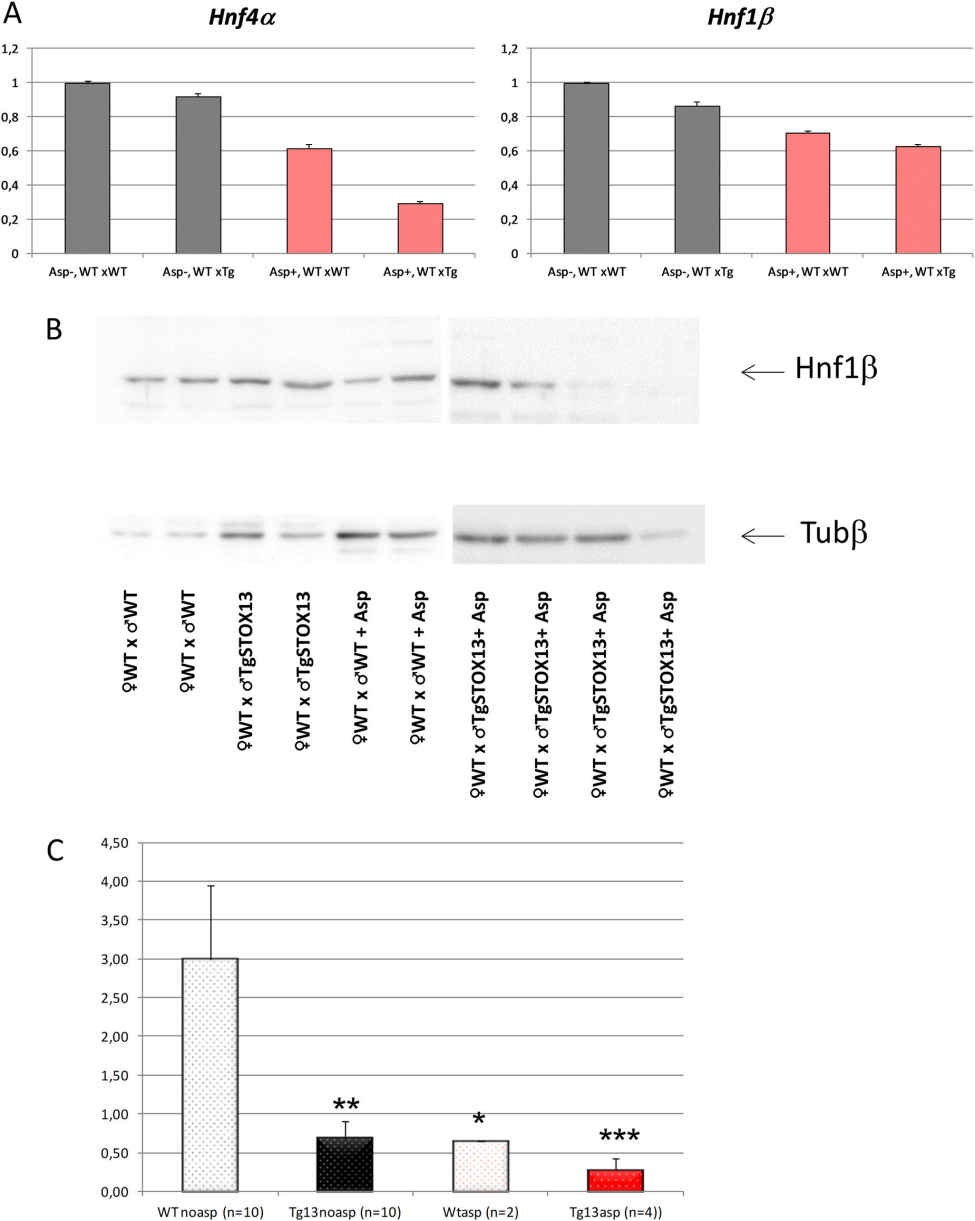
Expression levels of Hnf at the RNA (HNF4α and HNF1β) and protein level (HNF1β). **a** *Hnf4*α and *Hnf1*β expressions are modified in the placentas by Aspirin (in red) and by STOX1 overexpression (in large oblique hatches). The data were collected from the microarray analysis E-MTAB-1970. **b** Hnf1β is also deregulated at the protein level as quantified by Western Blot (presented is a representative sample of the four types of placentas analyzed). The quantification from all the placentas analyzed (26) is presented in **c**. The statistical analysis was performed by one-way ANOVA followed by Dunnett post-hoc *t*-test (*, **, *** stands for *p* < 0.05, *p* < 0.01 and *p* < 0.001, respectively)

Statistically the data were analyzed by two-ways ANOVA, the factors being ‘aspirin treatment’ and ‘STOX1 overexpression’ (Fig. 4a). We could observe a significant deregulation of *Hnf1β* and *Hnf4α* by aspirin (*p* = 0.007 and 0.0002, respectively). STOX1 overexpression tended to down-regulate both genes, but only *Hnf4α* was significantly reduced (*p* = 0.013). In this case, there was a significant interaction effect between the two factors, aspirin and STOX1 (*p* = 0.009). This interaction indicates a synergistic effect of STOX1 and aspirin in modifying the level of *Hnf4α* mRNA. In sum, STOX1 tends to down-regulate *Hnf4α* gene expression, and aspirin potentiates this effect.

To analyze this regulation at the protein level we prepared protein extracts from WT and transgenic placentas from pregnant mice treated with aspirin or untreated. Western Blot gave a clear signal only for *HNF1β* (Fig. 4b) Without aspirin, STOX1A was able to decrease about four-fold the protein level of HNF1β when comparing WTnoasp vs. Tg13noasp (Fig. 4c). Aspirin alone also tended to decrease the level of HNF1β (~four-fold, between WTnoasp and WTasp). The effect of STOX1A and aspirin appeared synergistic, with a decrease of HNF1β expression reaching 12-fold. In sum, STOX1A down-regulates HNF1β, and these modifications appear stronger at the protein level than at the mRNA level. These results led us to hypothesize that the transcriptome profile in the mouse placentas are due, at least partially to an alteration of the expression of HNF factors, leading to the deregulation of genes of the coagulation cascade.

### Expression of HNF1 targets in the human placenta in aspirin-treated patients

**Fig. 5:**
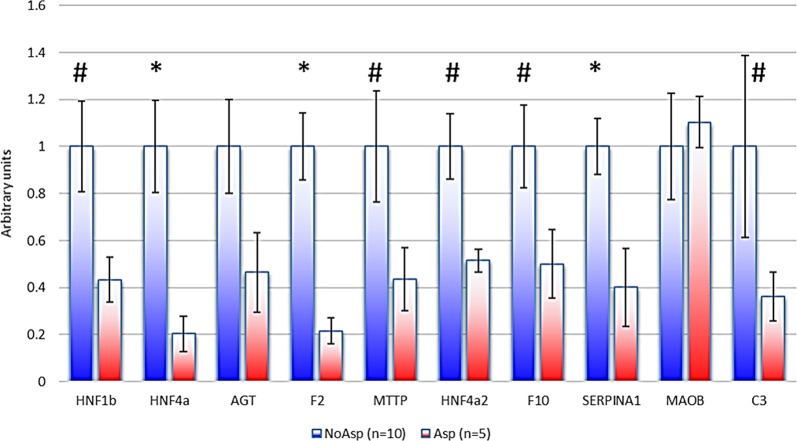
Expression of HNF1β and HNF1β-target genes is reduced in human placentas under aspirin treatment (*p   <  0.05, ^#^*p*  <  0.10). At the 0.05 threshold, only HNF4a, F2, and SERPINA1 were significantly reduced

Fifteen placental samples from preeclamptic patients were collected (5 from women that were treated by Aspirin, and 10 untreated). We studied the expressions of 9 genes by qRT-PCR (Fig. 5). Excepted MAOB, all the genes were down-regulated, and despite a certain heterogeneity between samples, *HNF4*α, *F2*, and *SERPINA1* were significantly down-regulated (*p* < 0.05), while four other genes (*HNF1β*, *MTTP*, *F10*, and *C3*) presented a trend towards decreased expression (*p* < 0.10). Due to the inter-placenta heterogeneity, AGT down-regulation did not reach statistical significance, even as a trend. Thus, aspirin effect at low doses impacts genes of the coagulation and of the complement cascade in a similar fashion in humans and in mice.

### The expression of HNF1 targets is modified by STOX1A in a trophoblast cell model

**Fig. 6:**
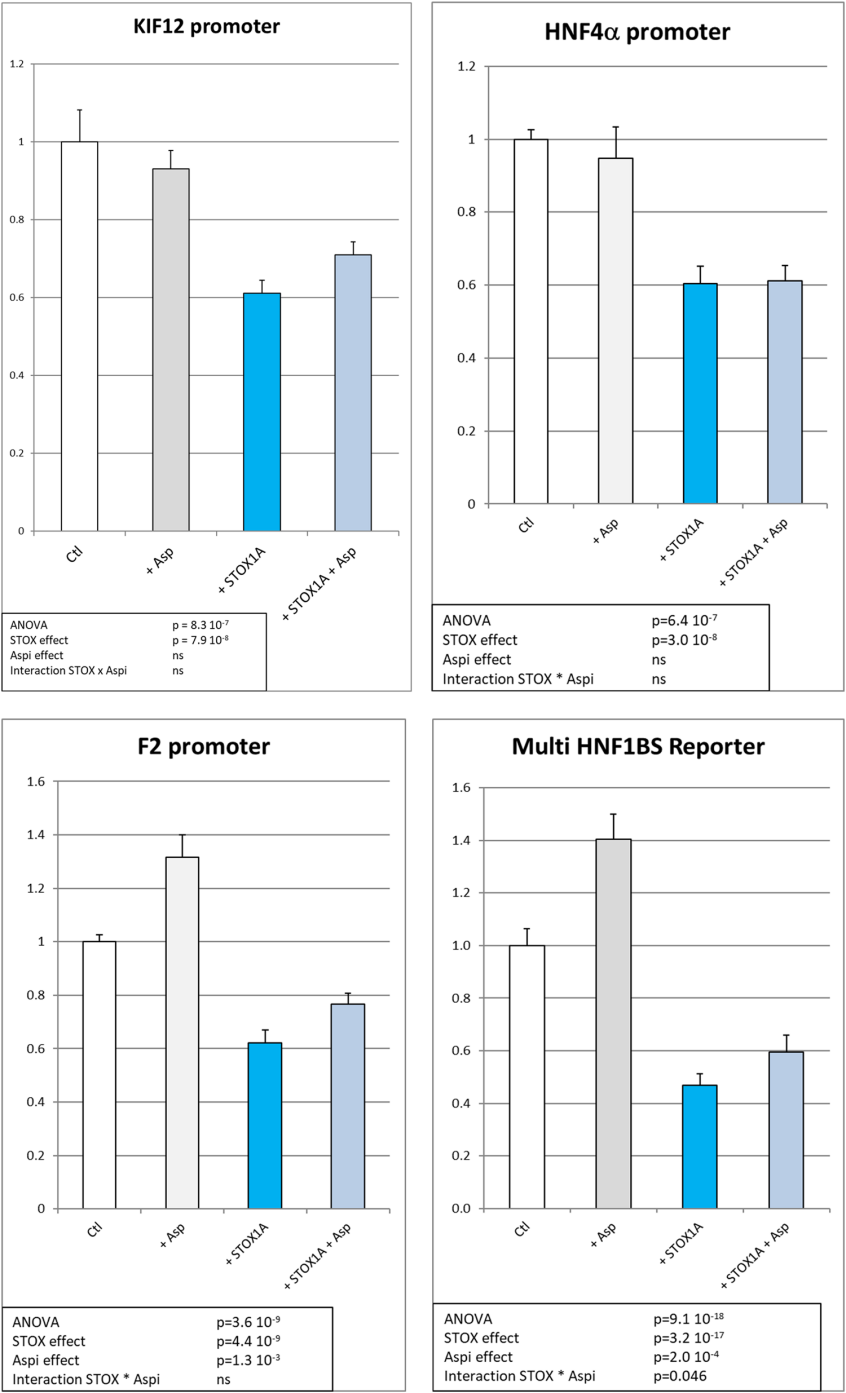
Luciferase assays in JEG-3 cells for the four reporter plasmids encompassing the luciferase gene under the control of promoters that are bound by HNF factors, the KIF12 promoter, the HNF4a promoter, the F2 promoter, and a synthetic promoter encompassing a 10 x polymer of the HNF1-binding site. In white are the control samples (transfection of the promoter with an empty expression vector). In gray, aspirin was added to the cells in culture. In turquoise blue, STOX1A was overexpressed, while in gray-blue, aspirin was added to the cell culture medium at 10 µg/ml. The experiment was performed as six independent transfections of four to six replicates for each construction, each transfections being performed in parallel with a Renilla encoding vector. The data presented are the ratio of the Luciferase/Renilla activity, using the DualGlo Luciferase protocol. The statistical analysis was performed by two-ways ANOVA followed by post-hoc Student–Newman–Keuls *t*-tests. The statistical results are summarized in a frame in each analysis (ns stands for ‘non-significant’)

To understand the mechanism of regulation of HNF1β targets (including HNF4α) by STOX1 and aspirin, we analyzed their effect in the JEG-3 cells, a proxy for human extravillous trophoblasts (EVTs). We used four reporter constructions encompassing HNF1β-binding sites, one including 320 bp of the human KIF12 (kinesin family member 12) promoter^12^, controlling the expression of Luciferase (KIF12 promoter in Fig. 6), two constructions corresponding to the promoter of F2 and the promoter of HNF4α, and finally one encompassing 10 repeats of the basic HNF1β-binding site cloned in front of the KIF12 basal promoter of 143 bp (HNF1BS Reporter in Fig. 6) in the Luciferase PGL-3 reporter plasmid (Promega). Transfections were performed in human JEG-3, as four replicates in six independent experiments, and resulted in very reproducible data. ANOVA analysis indicated that there was a significant down-regulation of the HNF targets following STOX1A overexpression on the four different promoters. We could also identify a significant but mild effect of Aspirin on two different promoters (F2 and multi-HBF1BS reporter).

The multi-HNF1BS promoter, where the HNF-binding site is polymerized 10 times, showed the strongest down-regulation of the expression by STOX1A overexpression (2.5 fold, *p* = 9.1 10^−18^), while aspirin tended to increase mildly but significantly the HNF Reporter expression level (+15–20%, *p* = 2 × 10^−4^). Overall, STOX1 overexpression reduces the expression of genes that are HNF targets. Aspirin marginally compensates this down-regulation.

## Discussion

### HNF proteins appear as hub proteins of aspirin action in the placenta

Aspirin at low doses, has a protective effect against PE^4,13,14^. Administered early in gestation (before 16 weeks) aspirin could be very potent to protect against PE, especially in pregnancies at risk^6,7,9^. However, the molecular effects of aspirin in the context of PE remain unexplored. Aspirin could fight the deleterious effects of PE on the maternal vascular endothelium, but aspirin could also act directly on the placenta. In the present study we investigated the later possibility.

Using a mouse model where PE is induced by the overexpression of STOX1A in the foeto-placental unit, and rescued by low-dose aspirin treatment^10^, we analyzed gene expression in the normal and preeclamptic placenta with or without aspirin treatment. We tested two hypotheses: either (i) aspirin modulates the expression of the same genes than STOX1A in the opposite direction, or (ii) it acts on different genes. Here, we show that the second hypothesis is true.

In the transgenic placentas, aspirin down-regulates genes involved in the complement and coagulation cascades, as well as genes involved in lipid transport and metabolism, which are not specifically altered by STOX1A overexpression alone. To understand how these genes are deregulated by aspirin, we analyzed their promoters by bioinformatics and found that the most down-regulated genes are strongly enriched in HNF proteins binding sites. Of the four HNFs, HNF4α and HNF1β were expressed and significantly down-regulated at the mRNA level in the transgenic placentas from mice treated with aspirin. This is true also for HNF1β at the protein level (interestingly HNF4α is known as a transcriptomic target of HNF1β^15^), suggesting that the down-regulation of the latter could explain that of the former. HNF1β expression has also been associated to coagulation via a three-fold increased risk of thrombosis in tumors^16^. In the context of coagulation, HNF1β-binding sites are present in the promoter of the C-reactive protein (CRP), an anticoagulant molecule synthesized in the liver^17^. More recently, exome sequencing revealed associations between the level of CRP and HNF1α genetic polymorphisms^18^. Here we show a down-regulation of several HNF factors by STOX1A overexpression in JEG-3 human cells (GEO dataset GSE13475^19^) as well as in the mouse placentas (EMBL accession number E-MTAB-1970^10^), but clearly these modifications are not strong enough to compensate for the noxious effects of STOX1A overexpression leading to PE. Consistently, we found that HNF1β targets were all down-regulated by STOX1A overexpression (Fig. 6). The mild effect of aspirin observed in vitro for two promoters, could be due either to the fact that adding aspirin on culture cells is quite dissimilar than to have it given in the drinking water in vivo, or to the fact that trophoblast cells could be less sensitive to aspirin than the placenta in vivo, although several publications report effects of aspirin in trophoblast cells in culture^20^.

The HNF transcription factors (especially HNF1β and HNF4α) regulate the expression of a large number of coagulation genes, such as prothrombin (encoded by the *F2* gene), FVII, FVIII, FIX, FX, FXI, FXII, protein S, protein Z, and antithrombin^21^. HNF4α knock-out mice or short-interfering RNA (siHNF4α) injected mice show a dramatic down-regulation of many genes of the coagulation cascade in the liver^22^. Beside coagulation, HNF4α positively regulates the expression of several components of the complement, including the critical components C3 and CFB^23^. Both are significantly down-regulated in the transgenic placentas by aspirin treatment in our experiment. HNF4α regulates also the expression of key genes in xenobiotic metabolism, bile acid synthesis and conjugation, lipid homeostasis, and gluconeogenesis^11,24^.

A question raised by our study is how aspirin treatment decreases HNF expression in the placenta. Clearly, as mentioned above, aspirin has no major transcriptional effect in JEG-3 cells, contrary to what is seen in the whole placenta, suggesting either that the regulation is different in other cells, or that other signaling pathways are at work in this context. In addition, HNF4α is known to be post-transcriptionally regulated by miR-24 and miR-34a, and a recent study using HepG2 cells transfected with miR-24 and miR-34a has shown a decrease not only of HNF4α but also of F10, F12, SERPINC1, PROS1, PROC, and PROZ transcripts levels^25^. The same study reported positive and significant correlations between the levels of HNF4α and several hemostatic factors in human liver samples. Interestingly, p53 can directly regulate the expression of the miR-34 family members^4,26–28^. Aspirin is known to be able to acetylate p53 which leads to increased protein stability and binding to promoters^29–31^. A recent study reports that increased p53 expression and miR-34a is responsible for the observed HNF4α down-regulation in non-alcoholic fatty liver disease^5^. Also, in non-alcoholic steatohepatitis (NASH) patients, the HNF4α protein is almost absent, leading to a drastic reduction of all liver functions.

Overall, we observed in the transgenic placentas treated with aspirin a significant reduction in the expression of HNF4α, and of several of its target genes involved in coagulation, complement cascades, lipids transport, and homeostasis. We propose that at least partly, aspirin action in the placenta occurs through the down-regulation of HNF4α, acting as a hub gene.

### Coagulation regulation in pregnancy

Aspirin treatment significantly down-regulated the expression levels of KNG1, FGB, F10, and F2, all pivotal actors of clot formation. Pregnancy is associated with a pro-coagulant state induced by increased coagulation factors and reduced anti-coagulation factors^32,33^. This pro-coagulant state is enhanced in the PE placentas, with disseminated intravascular coagulation being one of the hallmarks of PE^34,35^. Fibrinogen gamma (FGG), fibrinogen beta (FGB), and kininogen (KNG1), all these involved in the formation of the fibrin clot, are up-regulated in both serum and placental tissues from preeclamptic women^36,37^. In cultured trophoblasts, thrombin (F2) enhances the production of the anti-angiogenic factor sFLT1. Thus, thrombin could promote PE by interfering with local vascular transformation^38^. Thrombin generation is regulated by tissue factor (TF), which is encoded by F3 and expressed constitutively in the trophoblasts. In patients with PE, the maternal levels of TF, of thrombomodulin (THBD) and type 1 plasminogen activator inhibitor (SERPINE1) are elevated compared to normal pregnancies. Moreover, the expression of AnnexinA5 (ANXA5) in trophoblasts is reduced in the PE compared to non-pathological placentas^39^. ANXA5 plays a role in the inhibition of blood coagulation by binding to anionic phospholipids, thereby inhibiting aggregation and/or down-regulating the cell surface presentation of TF^40,41^. Therefore, PE placentas offer favorable environment for the production of thrombin, which can potentially damage the placenta by inducing fibrin deposition, inflammation, and sFLT1 expression with both local and systemic anti-angiogenic effects^42^.

### Complement regulation in pregnancy

Our study reveals that low dose aspirin treatment significantly down-regulates the placental expression of C3 and of complement factor B (CFB). CFB is cleaved by factor D into two fragments: Ba and Bb. Bb, is a serine protease, which combines with complement C3b to generate the C3 or C5 convertase. Down-regulation of these two critical components could prevent or alleviate placental complement activation in the STOX1 model of PE. Other animal models of PE, suggest that inhibition of the complement cascade could prevent most of the symptoms of the disease. Mice infused with AT1-AA show enhanced complement activation in the placenta and kidney with placental damage and PE symptoms. In this model the C3a receptor antagonist SB290157 prevents the increase in blood pressure, proteinuria, fetal growth restriction, and increase in sFlt-1 suggesting an important role for C3a in the development of the preeclamptic symptoms and in the systemic endothelial dysfunction^43^. In another mouse model of PE (DBA/2-mated CBA/J) the blockade of placental complement activation throughout pregnancy by the administration of the C3 inhibitor CR2-Crry prevented oxidative stress and placental dysfunction, as well as proteinuria and other renal pathologic features of PE. Thus, local blockade of complement activation at the maternal–fetal interface rescues the development of the PE-like symptoms^44^. In humans, it is known that pregnancy involves complement activation by C3 and C5, which is associated with pregnancy loss, showing that complement activation can adversely affect gestation. In severe PE, C5a and soluble C5b-C9 are most specifically elevated, suggesting that activation of the terminal pathway is a critical feature of severe disease^45–47^. In the placenta, complement activation localizes to injured villous trophoblast^48,49^, consistently with a hyper-activation in the context of PE. The complement cascade is divided into three traditional activation pathways (Supplementary Fig. 1), which converge to activate C3 and C5 leading to the formation of the membrane attack complex in the membrane. The coagulation and complement cascades are connected, since thrombin (F2) directly cleaves complement C3 and C5 to generate the anaphylatoxins C3a and C5a^50^.

### Lipid transport regulation in pregnancy

In the STOX1 mice, aspirin reduced the placental expression of many genes involved in lipid transport and metabolism: the apolipoproteins (APOA1, APOA2, APOA4, APOB, APOC2, APOC3, APOE, APOH, and APOM), the microsomal triglyceride transfer protein (MTTP), the LDL receptor-related protein 2 (LRP2), the fatty acid-binding protein 2 (FABP2), and the phospholipase A2 group XIIB (PLA2G12). The APOB and MTTP, known to be expressed by the syncytiotrophoblast and placental stromal cells, are essential components of lipoproteins assembly and transport. The APOB receptors, LRP2 (down-regulated in our model by aspirin treatment, Fig. 3) and LDLR are expressed by the cytotrophoblasts, the stromal cells of the chorionic villi, and the fetal endothelial cells^51^. Sub-endothelial retention of APOB-containing lipoproteins is an initiating event in atherogenesis, and high plasma levels of APOB is considered a risk factor for atherosclerosis, whereas low levels are supposed to provide protection. Thus the decrease in APOB and MTTP induced by low dose aspirin treatment could contribute to reduce both the pro-coagulant and atherogenic risk locally in the placenta. The transgenic placentas treated with low dose aspirin show also significant down-regulation of the vitamin D receptor (VDR) and in particular of the vitamin D-binding protein (GC), which is the major serum protein involved in the transport of vitamin D sterols. Vitamin D deficiency or insufficiency is thought to be common among pregnant women. Also, vitamin D supplementation during pregnancy seems to offer some protection against adverse pregnancy outcomes including PE. Thus, the decreased expression of both VDR and GD induced by aspirin could be a potential adverse effect, and suggests that vitamin D supplementation could be associated with low-dose aspirin treatment for PE.

## Material and methods

### Animal experiments

Animal experiments were previously described in ref. ^10^. Placentas were collected at 16.5 days post-coitum from female mice crossed either with WT or STOX1 transgenic males, and either treated or notwith aspirin during their pregnancy, as described previously^10^. Each hybridization was carried out in duplicate on Nimblegen-Roche microarrays using RNA from three placentas from different mice. The microarray and the details of the procedures (Array Express, E-MTAB 1970) are presented in ref. ^10^.

### Human ethics

Patients involved in this study, were informed about the study in a dedicated visit, and have given their informed consent to collect and use their biological samples. All protocols have been approved by the local Ethics Committee (No. CPP Am5724-1-COL2991; CODECOH No. DC-2012-1645).

### Cell culture, cloning, and transfections

JEG-3 cells were grown in Dulbecco’s modified Eagle’s medium Glutamax (DMEM, Life TechnologiesTM) with 10% of heat-inactivated fetal calf serum (FCS, Life Technologies) and 1% penicillin/streptomycin (S/P) at 37 °C in the presence of 5% CO_2_ and 20% O_2_. The cloning of the prothrombin (F2) promoter region was carried out using the primers 5′-ACGCATGGTACCCTGCTCTTTGTCCCTCTGTCC-3′ and 5′-CTCGAGAAGCTTCTGTGCACAAGGCTACACAG-3′, and the cloning of HNF4α P2 promoter was carried out using the primers 5′-GGTACCGAGCTC-CAGCAGGTTGAATTTAGAATGG-3′ and 5′-CTCGAGAAGCTTCCTGTCCAGTCTTCCCCCAG-3′. The PCR was carried out using the KAPA HiFi polymerase (Clinisciences) following the manufacturer’s indications, digested by *KpnI* and *HindIII* (New England Biolabs), and cloned in the PGL3 enhancer previously digested by the same enzymes (the small linker having been removed by ammonium acetate precipitation). The F2 promoter fragment corresponds to the positions 4096–5114 of the reference sequence NG_008953 (1018 bp) and the HNF4α promoter fragment corresponds to the positions 4106–5083 (977 bp) of the reference sequence NG_009818. Clones were sequenced and validated. Cells were seeded in 24-well plates the evening before transfection at 50% confluence, in order to reach ~75% the day after. The cells were transfected jointly with three plasmid constructions: pCMX STOX1A (400 ng) + Renilla (pRL-RSV, 10 ng) + either pGL3-KIF12 (390 ng), or pGL3-Multi HNF1BS Reporter (which corresponds to the HNF1β-binding site multimerized 10 times ligated to the minimal KIF12 promoter, 390 ng), or pGL3-F2 (390 ng) or pGL3-HNF4α (390 ng), using LipofectamineR2000 (Life Technologies™). The controls were identical except that the pCMX STOX1A vector was substituted by a pCMX-Empty vector. When aspirin was used, the cells were treated at a concentration of 10 µg/ml in the medium^20^, changed every 24 h from the day after transfection. Cell lysis was performed 4 days after transfection: after removal of medium and washing with DPBS (Life TechnologiesR), 100 µl of passive lysis buffer 1× (Promega) was added in each well and the plates were rocked during one hour. Then the lysate was transferred into a fresh tube. Transcriptional activity was assessed by the Dual-Luciferase Reporter Assay System (Promega) in 96-well plates according to the manufacturer’s instruction (50 µl instead of 100 of each reagent was added). Luminescence was measured using a Berthold Centro XS3 LB960 luminometer. The observed firefly’s activity was divided by the Renilla’s activity and the mean values of the replicates were calculated.

### Microarrays and bioinformatics

The microarray was made available with the EMBL-EBI accession number E-MTAB-1970. Twelve samples were hybridized on a Nimblegen Mouse Expression Array MM8_60mer. Each of them was from a RNA mix (each RNA with a RIN > 8, as evaluated by Agilent 2100 Bioanalyzer) of three placentas from three different pregnancies. Previously^10^, the effect of aspirin was not analyzed, and it is the focus of the present study. The complete dataset was first analyzed using Arraymining^52^, in order to produce a heatmap drawn from the 500 most significant genes (Fig. 1, feature selection method: eBayes). Webgestalt (http://www.webgestalt.org/option.php) was used to identify cascades that were massively deregulated in this gene list that allowed identifying significant KEGG pathways. The network analysis was carried out by submitting the list of 500 genes to String (http://string-db.org/), with the option ‘multiple names’^53^. Then the structure of the network obtained was saved and exported to Cytoscape (http://www.cytoscape.org/) a platform originally developed in 2003^54^. The sub-network composed of the down-regulated genes (Fig. 2a) was then analyzed using iRegulon^55^, a tool enabling to identify common-binding sites for transcription factors in a series of promoters.

### RNA preparation and RT-qPCR analysis from human placentas

High-quality RNA samples from 15 patients (5 under low-dose aspirin treatment and 10 not treated) were prepared from placental tissues collected at three different places from placentas obtained from C-section. After thorough rinsing with PBS and collection in TriZol (Invitrogen), the sample were frozen at −70 °C. RNA was extracted after grinding with a metal bead, adjunct of 1/5 volume chloroform, centrifugation, and precipitation of the upper phase with 0.8 vol of isopropanol, salt washing with ethanol 70%, and resuspension in an adequate volume. qRT-PCR was carried out from 2 µg total RNA, using classical protocols from Invitrogen and a MMLV cDNA kit synthesis. cDNA levels were quantified using primers, identified using the Harvard Website https://pga.mgh.harvard.edu/primerbank/^56^. More specifically, the qPCR primers are summarized as Supplementary Table 1. SDHA, considered as a very stable calibrator for placental gene expression^57^ was used as calibrator in the 2^−ΔΔCt^ method, as described^58^.

### Western blot analysis and quantification

Cells were lysed in 50 mM Tris–HCl pH 7.5, 150 mM NaCl, 1% Triton X-100, 0.1% sodium dodecyl sulfate (SDS), 1 mM EDTA, and protease inhibitor cocktail. Thirty micrograms of protein, estimated by Bradford reactions (Sigma-Aldrich), were loaded for SDS–PAGE. After blotting, hybond ECL nitrocellulose filters were probed with primary antibodies, then with secondary antibodies. Detection was carried out by exposition to Amersham ECL Hyperfilm and quantification was performed using the ImageJ software.

### Statistical analysis

Significance was evaluated using one-way or two-way ANOVA followed by post-hoc tests (Student–Neumann test) between the different groups if there were more than two groups. In comparison between aspirin-treated and non-treated human placentas, Student's *T* tests were used. The Statist’XL add-in excel was used. A value of *p* < 0.05 was considered significant.

## Supplementary information

Aspirin deregulated genes

Figure S1

Primer list

Supplemental Material File #1
