# Supplementary figures and images for: Low-dose aspirin protective effects are correlated with deregulation of HNF factor expression in the preeclamptic placentas from mice and humans
Source: Cell Death Discov. 2019 May 10;5:94. doi: 10.1038/s41420-019-0170-x (PMC6510804; doi:10.1038/s41420-019-0170-x)

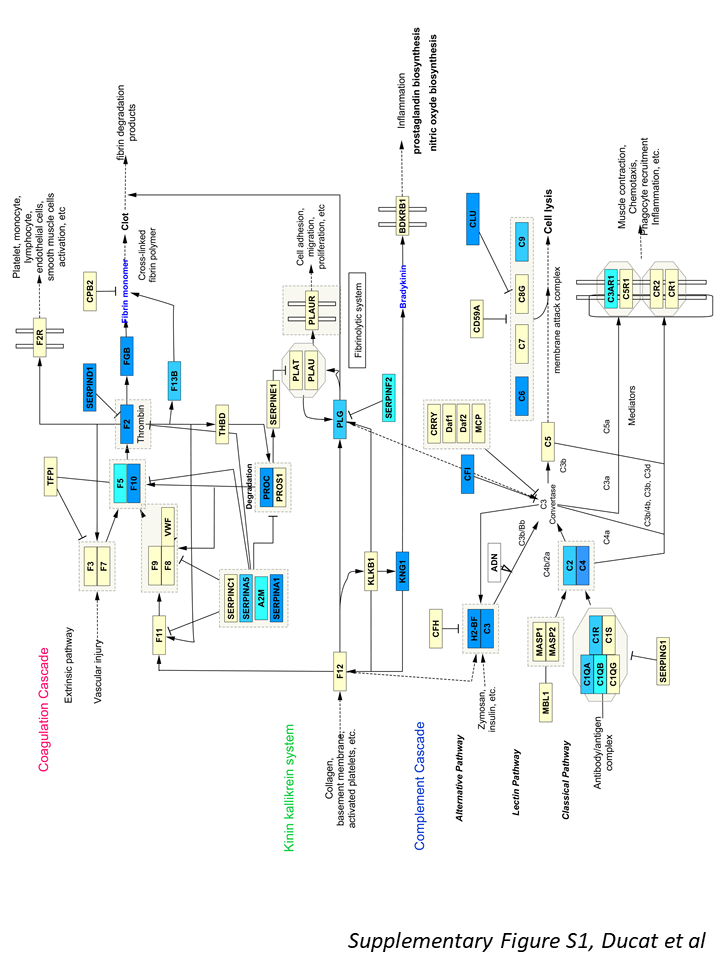

Supplement: Supplementary file 2 — Figure S1 [file 41420_2019_170_MOESM2_ESM.tif]

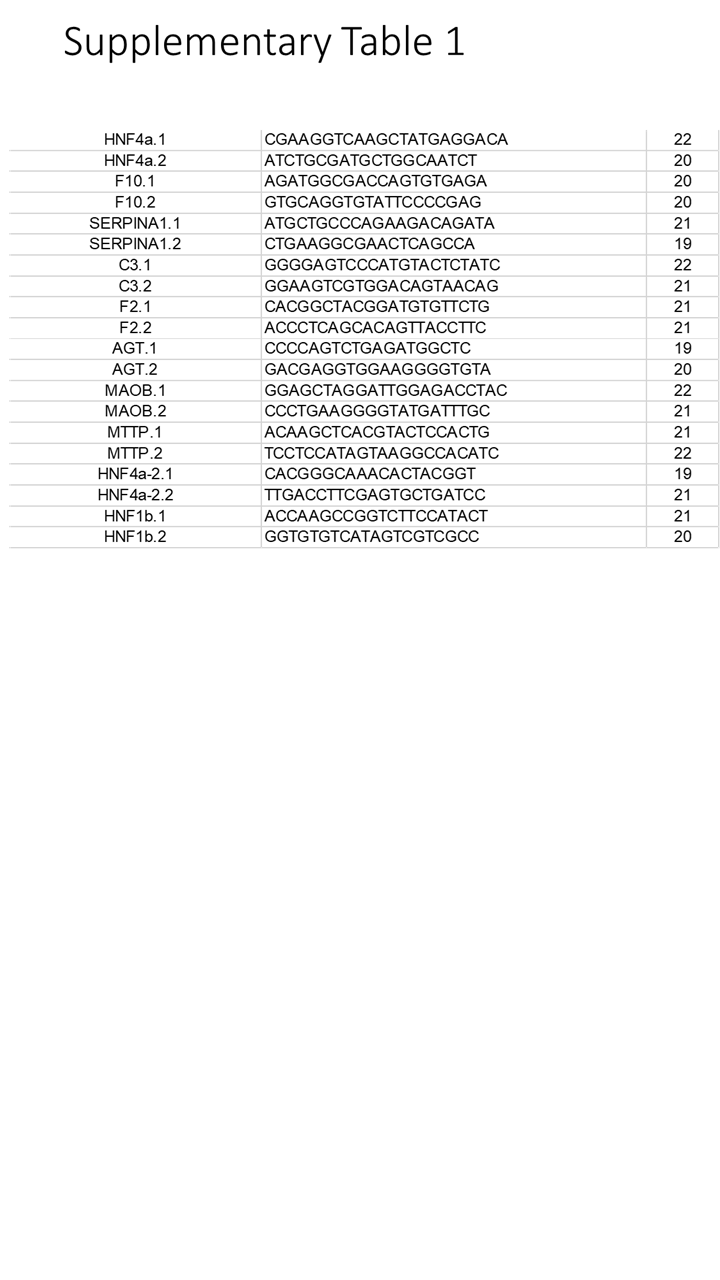

Supplement: Supplementary file 3 — Primer list [file 41420_2019_170_MOESM3_ESM.tif]
