# Supplementary material for: Low-dose aspirin protective effects are correlated with deregulation of HNF factor expression in the preeclamptic placentas from mice and humans
Source: Cell Death Discov. 2019 May 10;5:94. doi: 10.1038/s41420-019-0170-x (PMC6510804; doi:10.1038/s41420-019-0170-x)
Supplement: Supplementary file 4 — Supplemental Material File #1 [file 41420_2019_170_MOESM4_ESM.docx]

**Supplementary Figure Legend**

**Figure S1.** Description of the Coagulation and Complement Cascade with the genes modified by aspirin treatment in the mouse placentas. All the down-regulated genes are colored in blue.
